# Cross-attention guided multi-modal network for breast ultrasound diagnosis incorporating objective clinical semantics

**DOI:** 10.3389/fmed.2026.1802570

**Published:** 2026-06-09

**Authors:** Qin Sun, Xiaoman Wu, Ju Chen, Yuhang Zhang, Chao Zhou, Zheng Zhu

**Affiliations:** Department of Ultrasound Diagnostics, The First People’s Hospital of Taicang, Suzhou, Jiangsu, China

**Keywords:** breast ultrasound, computer-aided diagnosis, cross-attention, diagnosis, multi-modal learning

## Abstract

**Introduction:**

Breast ultrasound diagnosis is significantly constrained by operator subjectivity. While Deep Learning shows promise, existing models often neglect the structured morphological semantics essential for radiological reasoning.

**Methods:**

We propose Cross-Attention Guided Network (CGA-Net), a multi-modal framework that fuses visual data with objective clinical descriptors via a cross-attention mechanism. Specifically, clinical features—such as shape and margin—act as semantic queries to dynamically highlight pathological regions.

**Results:**

Validated on 252 patients using a rigorous Out-Of-Fold (OOF) prediction strategy to prevent data leakage, the CGA-Net trained from scratch demonstrated the most balanced clinical utility, yielding a robust OOF AUC of 0.905 with the highest overall accuracy (0.857) and an optimally balanced specificity (0.831), while maintaining excellent sensitivity (0.898). Furthermore, a pre-trained version of CGA-Net achieved a peak overall ranking AUC of 0.915. Both multi-modal configurations substantially outperformed the robust clinical-only baseline (0.890) and the image-only baseline (0.795).

**Conclusions:**

This suggests that while transfer learning aids general feature extraction, strong cross-modal semantic guidance alone is highly effective at reducing false positive diagnoses and optimizing practical clinical thresholds. Attention map visualization confirmed that the model aligns closely with expert focus on tumor periphery. CGA-Net offers a robust, interpretable, and data-efficient “second opinion” tool to reduce diagnostic variability.

## Introduction

Breast cancer remains the most prevalent malignancy and a leading cause of cancer-related mortality among women worldwide (1). Early and accurate diagnosis is pivotal for improving survival rates, with breast ultrasound (US) serving as a primary screening modality due to its non-invasiveness, cost-effectiveness, and radiation-free nature (2). However, the diagnostic accuracy of ultrasound is effectively constrained by cognitive variations in integrating these features. While the BI-RADS lexicon provides a standardized terminology for describing lesions, including shape and margin, the reasoning process required to map these morphological descriptors to a final diagnostic conclusion remains operator-dependent. This inherent subjectivity often leads to significant inter-observer variability, driving up false-positive rates and resulting in unnecessary biopsies. To address these challenges, Computer-Aided Diagnosis (CAD) systems, particularly those based on Deep Learning (DL), have emerged as powerful tools to assist clinicians in distinguishing benign from malignant lesions (3).

In recent years, Convolutional Neural Networks (CNNs) have achieved remarkable success in breast ultrasound analysis, often matching or even surpassing human experts in classification tasks (4). Despite these advancements, the majority of existing AI models operate as purely visual systems that process raw images in isolation, neglecting rich structured clinical information, specifically the standardized BI-RADS descriptors routinely utilized by radiologists during diagnosis (5). While visual features are essential, they are susceptible to image artifacts and noise. In contrast, clinical semantic priors, such as “spiculated margin” provide robust, high-level diagnostic evidence that is invariant to image quality. Consequently, pure vision-based models often function as “black boxes,” lacking the ability to align their decision-making process with established pathological criteria, which limits their trustworthiness in clinical practice (6).

Recognizing this limitation, recent research has shifted toward multi-modal learning, aiming to fuse visual data with clinical context (7). However, current approaches typically rely on naive fusion strategies, such as simple concatenation of feature vectors from different modalities (8). These methods fail to model the intricate interactions between visual patterns and semantic descriptions. For instance, a clinical description of an “irregular shape” should actively guide the model to focus on the lesion’s boundary, rather than merely being appended as a passive variable. Furthermore, most medical AI frameworks heavily rely on transfer learning from large-scale natural image datasets, most notably ImageNet (9). While effective for data-scarce tasks, this paradigm may be suboptimal when strong domain-specific semantic guidance is available, as the generic features learned from natural images may not align with the subtle, texture-dependent characteristics of ultrasound lesions (10, 11).

To bridge these gaps, we propose CGA-Net, a multi-modal framework designed to emulate the cognitive reasoning process of radiologists. Rather than proposing a fundamentally new mathematical architecture, the core methodological contribution of CGA-Net lies in its translational design: it leverages well-established cross-attention mechanisms to explicitly mimic the deductive reasoning of a radiologist, using structured clinical semantics as active queries to guide visual feature extraction. This allows the model to dynamically re-weight the visual feature map and “look” for specific pathological signs, such as posterior shadowing, guided strictly by clinical priors. Furthermore, our results reveal a crucial insight into model optimization: evaluated under a rigorous OOF protocol, we demonstrate that while large-scale pre-training maximizes the overall ranking metric (AUC 0.915), CGA-Net trained from scratch—driven purely by these semantic queries—achieves the best-balanced clinical performance, yielding the highest accuracy and specificity. By strictly excluding subjective assessment scores to prevent data leakage, this study presents a robust, interpretable, and data-efficient solution that aligns algorithmic attention with pathological truth, offering a reliable “second opinion” for clinical diagnosis.

## Materials and methods

### Datasets and data acquisition

To develop and validate the proposed diagnostic framework, a comprehensive experimental setup utilizing both internal and external cohorts was constructed. The primary internal validation was conducted on the Breast Examination Assessment and Sharing of Tumors (BrEaST) dataset, accessed via The Cancer Imaging Archive (TCIA) (12). This dataset uniquely provides paired raw ultrasound images and structured clinical reports containing morphological descriptors. A total of 256 patients were initially screened, and their baseline clinical characteristics were recorded. After excluding 4 patients due to missing paired raw ultrasound images, a final study cohort of 252 patients (98 malignant and 154 benign) was curated to ensure complete multi-modal entries for the network training. It is important to note that each patient entry in this finalized cohort corresponds to exactly one representative ultrasound image paired with its structured clinical report. No multiple images or varying views from the same patient were included, ensuring a strict 1:1 patient-to-image ratio. To investigate the impact of domain-specific transfer learning, a large-scale external pre-training pool was additionally aggregated by harmonizing two public datasets: BUS_UC (13, 14) and BUSI (15). Because these public datasets are purely image-based and lack the paired structured clinical reports required for our multi-modal fusion, this external cohort served exclusively as a source for pre-training the visual backbone, rather than for external validation.

### Data preprocessing and feature extraction

To ensure the model focused on the pathological lesion rather than background artifacts, an automated Region of Interest (ROI) extraction protocol was implemented. For each case, the tumor bounding box was derived from the binary mask, and the ROI was cropped with a fixed margin of 20 pixels to preserve contextual boundary information. All images were subsequently resized to 224 × 224 pixels and normalized using standard ImageNet mean and standard deviation values (16). To preserve the diagnostic integrity of lesion growth patterns, such as parallel versus non-parallel orientation, random geometric augmentations were explicitly excluded from the training phase. Furthermore, as per standard practice, the validation set remained entirely unaugmented, with the overall preprocessing pipeline strictly limited to deterministic operations, namely resizing and normalization.

For clinical data, a strict feature selection strategy was applied to prevent potential data leakage. Subjective assessment scores, such as BI-RADS assessment categories, and final diagnostic conclusions were explicitly excluded. Because BI-RADS categories represent the radiologist’s final subjective diagnostic synthesis, they act as a highly correlated proxy for the ground truth. Including them as input features would introduce label leakage, allowing the model to bypass the intended cross-modal feature integration. Furthermore, while BI-RADS categories are clinically meaningful, they were not utilized as target labels. Our framework was intentionally designed as a binary classification task (benign versus malignant) grounded strictly in biopsy-proven pathological outcomes. This approach ensures the model learns the objective pathological truth rather than a subjective radiological opinion, while also avoiding the severe class imbalance that would arise from stratifying our 252-patient cohort across multiple BI-RADS subcategories. Consequently, only objective morphological descriptors were selected as inputs, specifically: Shape, Margin, Echogenicity, Posterior Features, and Calcifications. These categorical features were processed via one-hot encoding, while patient age was normalized to the [0, 1] range. The final structured clinical vector (*v*_*clin*_ ∈ ℝ^32^) served as the semantic input for the network.

### Network architecture

**FIGURE 1:**
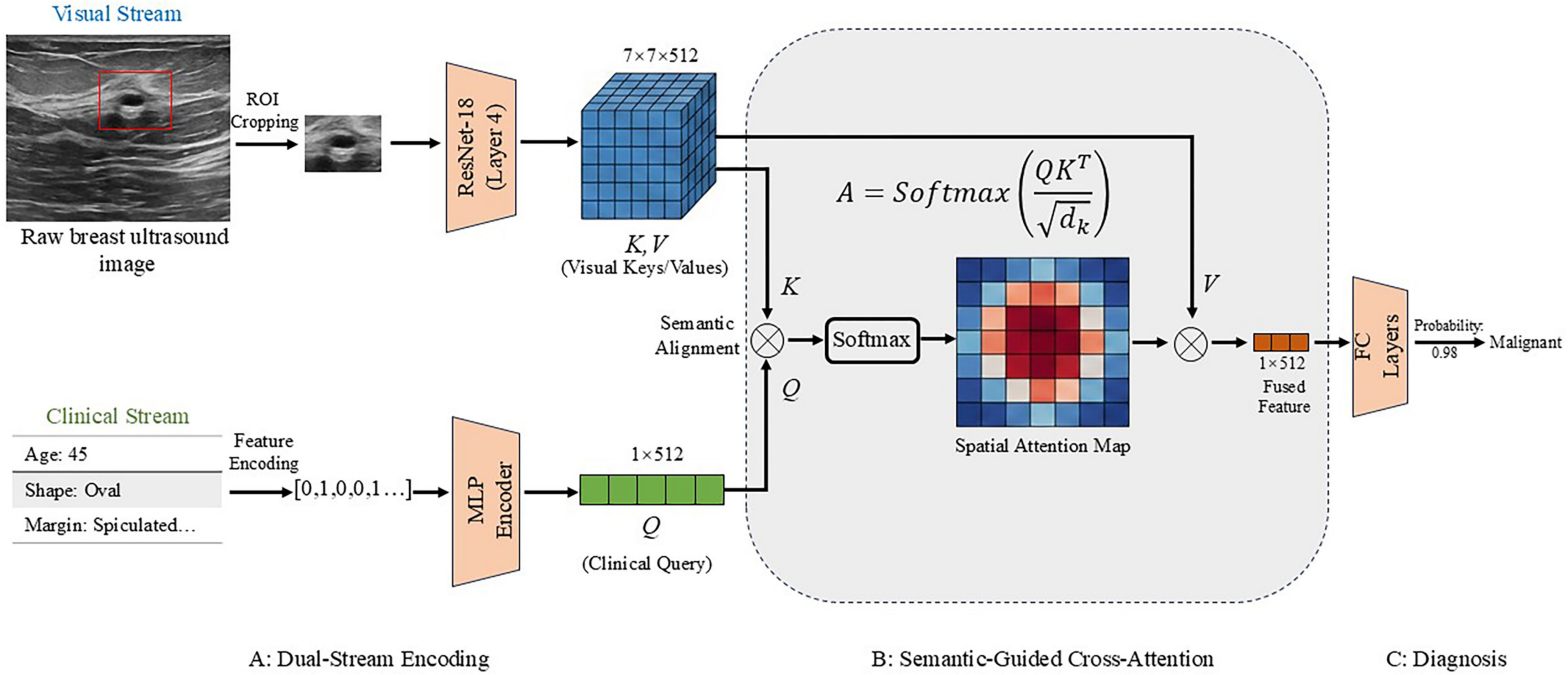
Architecture of the proposed CGA-Net.

The proposed CGA-Net architecture, as illustrated in Figure 1, integrates a Visual Encoder, a Clinical Encoder, and a Cross-Attention Fusion module to emulate the radiologist’s multi-modal reasoning process. A ResNet-18 backbone was adopted as the visual feature extractor to mitigate overfitting on the relatively small patient cohort. In medical imaging tasks with limited sample sizes, shallower networks like ResNet-18 are widely recommended over deeper architectures to prevent severe overfitting while maintaining robust feature extraction (17). To preserve spatial information, the network was truncated at the fourth residual block, discarding the final global average pooling and fully connected layers. This encoder maps the input image to a high-level spatial feature map. Simultaneously, structured clinical inputs are processed by a Multi-Layer Perceptron (MLP) acting as the Clinical Encoder. The MLP comprises three fully connected layers (dimensions: 32→128→256→512) designed to smoothly project the low-dimensional clinical vector into the 512-dimensional space required for stable cross-attention alignment. This gradual, hierarchical expansion is implemented to prevent the representation bottlenecks and training instability often encountered when mapping directly from low-dimensional inputs to high-dimensional latent spaces (18). Each layer is followed by Batch Normalization, ReLU activation, and Dropout (*p* = 0.3) to mitigate overfitting.

The core integration is achieved via the Cross-Attention Fusion Module. Inspired by established multi-modal transformer architectures, this module mathematically aligns spatial visual features with clinical semantics (19). To ensure dimensional compatibility, the spatial visual feature map extracted from the ResNet-18 backbone, denoted as *F*_*v*_ϵℝ^*H*×*W*×*C*^ (where spatial dimensions *H* = *W* = 7 and channel depth *C* = 512), is flattened into a sequence of *N* visual tokens *X*_*v*_ϵℝ^*N* = *C*^ (*N* = 49). Simultaneously, the clinical encoder projects the structured clinical vector into the same latent space. To generate the Queries (Q), Keys (K), and Values (V), the clinical vector and the flattened visual sequence *X_v_* are transformed via dedicated, learnable linear projection matrices (512×512). A single-head attention configuration was deliberately employed to produce a singular, cohesive 2D spatial attention map that directly reflects the overall clinical query. The cross-modal interaction is formally defined as:


$$A=\text{Softmax}\,\left(\frac{Q\,K^{T}}{\sqrt{d_{k}}}\right)$$

where *d*_*k*_ = *C* = 512 represents the dimension of the keys, and *A*ϵℝ^1×*N*^ represents the generated spatial attention map. This map dynamically quantifies the relevance of each of the 49 visual regions to the specific clinical query. The final multi-modal representation is computed as the context-weighted sum *F*_*fused*_ = *AV*. This results in a fused vector *F*_*fused*_ϵℝ^1×*C*^ that effectively filters out irrelevant background noise before passing into the final diagnostic layers. Specifically, the classification head is structured as a two-layer Multi-Layer Perceptron (MLP) comprising a linear layer mapping from 512 to 64 dimensions, followed by a ReLU activation, and a final linear projection to a single logit representing the malignancy probability.

### Experimental setup and training protocol

All experiments were implemented using Python 3.9.25 and PyTorch 2.8.0 on a single NVIDIA GeForce RTX 4060 GPU (20). The proposed CGA-Net was trained using 5-fold Stratified Cross-Validation. Because each case corresponds to a unique patient, this split was strictly performed at the patient level, entirely eliminating the risk of data leakage caused by same-patient images appearing in both training and validation sets. The optimization was performed using the standard Adam optimizer. To rigorously separate hyperparameter tuning from the final performance evaluation and prevent optimistic bias, hyperparameters (including a learning rate of 1 × 10^–4^, an L2 regularization penalty of 1 × 10^–4^, and a batch size of 16) were determined via empirical grid search conducted exclusively within the training folds. Once these optimal hyperparameters were established, they were fixed globally across all folds for the final aggregated OOF testing. Specifically, a relatively small batch size of 16 was selected as it introduces beneficial gradient noise, acting as an implicit regularization mechanism that enhances generalization on limited medical datasets (21). Furthermore, the conservative learning rate and L2 penalty were chosen to ensure stable convergence of the cross-attention weights and prevent overfitting during fine-tuning. The binary cross-entropy loss with logits was used as the objective function. Each fold was trained for 50 epochs, and the model with the highest validation AUC was saved for testing. For comparative analysis, a two-stage transfer learning strategy was also implemented, where the visual backbone was first pre-trained on the external cohort (BUSI + BUS_UC) before fine-tuning. Both the from-scratch and pre-trained versions of CGA-Net were systematically evaluated to analyze the nuanced trade-off between transfer learning and direct semantic guidance. Furthermore, we established a Clinical baseline constructed as a Multi-Layer Perceptron (MLP) with the same architecture as the clinical branch of CGA-Net. This model was trained solely on structured morphological features to quantify the diagnostic ceiling of objective clinical semantics.

### Statistical analysis

Statistical analysis was performed using Scikit-learn (v1.6.1) and SciPy (v1.13.1) libraries. For baseline characteristics, continuous variables, such as Age, were compared using the Mann-Whitney U test due to the non-normal distribution of the data, while categorical variables, such as Shape and Margin, were compared using the Chi-square test. Model performance was evaluated using AUC, Accuracy, Sensitivity, and Specificity. To strictly prevent data leakage and ensure an unbiased evaluation across the entire dataset, all performance metrics were calculated using an aggregated OOF prediction strategy. Specifically, the predicted probabilities for all 252 patients were generated exclusively by the models for which those respective patients were in the unseen validation fold. To assess the statistical robustness of these results, 95% confidence intervals (CI) were estimated using bootstrap analysis with 1,000 iterations strictly on the aggregated OOF predictions. Comparison between AUCs of different models was performed using the DeLong test (22). To evaluate the reliability and calibration of the model’s predicted probabilities, the Expected Calibration Error (ECE) and Brier score were calculated on the OOF predictions. Statistical significance was defined as a two-sided *P*-value < 0.05.

## Results

### Statistical analysis of patient demographics and morphological features

The initial clinical cohort comprised 256 patients, whose baseline characteristics and morphological features are detailed in Table 1 (including 4 cases lacking image data, annotated as “not applicable”). Following the exclusion of the 4 incomplete cases, the internal validation cohort strictly utilized for the multi-modal network evaluation comprised 252 patients, including 98 malignant cases and 154 benign cases. The mean age of the malignant group (60.0 ± 12.3 years) was significantly higher than that of the benign group (49.4 ± 14.0 years, *P* < 0.001). Statistical analysis revealed that these features possessed strong discriminative capability. Specifically, 93.9% (92/98) of malignant tumors presented an “irregular” shape, whereas the benign group was dominated by “oval” (58.2%) or “round” (8.9%) shapes (*P* < 0.001). Regarding tumor margins, 96.9% of malignant cases exhibited non-circumscribed characteristics (indistinct, angular, or spiculated), in sharp contrast to the 70.9% of benign masses with circumscribed margins (*P* < 0.001). Furthermore, posterior shadowing was observed in 32.7% of malignant cases compared to only 11.4% in benign cases (*P* < 0.001). These significant disparities provided a robust pathological basis for the proposed multi-modal fusion network.

**TABLE 1:** Baseline characteristics of the study population.

Characteristic	Total	Benign	Malignant	*P*-value
Age (years)	53.5 ± 14.3	49.4 ± 14.0	60.0 ± 12.3	<0.001*
Shape				<0.001*
Irregular	140 (54.7%)	48 (30.4%)	92 (93.9%)	
Oval	97 (37.9%)	92 (58.2%)	5 (5.1%)	
Round	15 (5.9%)	14 (8.9%)	1 (1.0%)	
Not applicable	4 (1.6%)	4 (2.5%)	0 (0.0%)	
Margin				<0.001*
Circumscribed	115 (44.9%)	112 (70.9%)	3 (3.1%)	
Not circumscribed—indistinct	52 (20.3%)	25 (15.8%)	27 (27.6%)	
Not circumscribed—angular and indistinct	23 (9.0%)	3 (1.9%)	20 (20.4%)	
Not circumscribed—speculated and indistinct	11 (4.3%)	2 (1.3%)	9 (9.2%)	
Not circumscribed—microlobulated and indistinct	11 (4.3%)	2 (1.3%)	9 (9.2%)	
Not circumscribed—microlobulated	10 (3.9%)	4 (2.5%)	6 (6.1%)	
Not circumscribed—speculated and microlobulated and indistinct	6 (2.3%)	2 (1.3%)	4 (4.1%)	
Not circumscribed—speculated and angular and indistinct	5 (2.0%)	1 (0.6%)	4 (4.1%)	
Not circumscribed—spiculated	5 (2.0%)	0 (0.0%)	5 (5.1%)	
Not applicable	4 (1.6%)	4 (2.5%)	0 (0.0%)	
Not circumscribed—speculated and angular and microlobulated and indistinct	4 (1.6%)	0 (0.0%)	4 (4.1%)	
Not circumscribed—angular	3 (1.2%)	2 (1.3%)	1 (1.0%)	
Not circumscribed—angular and microlobulated and indistinct	3 (1.2%)	1 (0.6%)	2 (2.0%)	
Not circumscribed—speculated and angular	2 (0.8%)	0 (0.0%)	2 (2.0%)	
Not circumscribed—angular and microlobulated	2 (0.8%)	0 (0.0%)	2 (2.0%)	
Echogenicity				<0.001*
Hypoechoic	148 (57.8%)	73 (46.2%)	75 (76.5%)	
Heterogeneous	57 (22.3%)	37 (23.4%)	20 (20.4%)	
Anechoic	15 (5.9%)	15 (9.5%)	0 (0.0%)	
Isoechoic	12 (4.7%)	12 (7.6%)	0 (0.0%)	
Complex cystic/solid	11 (4.3%)	8 (5.1%)	3 (3.1%)	
Hyperechoic	9 (3.5%)	9 (5.7%)	0 (0.0%)	
Not applicable	4 (1.6%)	4 (2.5%)	0 (0.0%)	
Posterior features				<0.001*
No	159 (62.1%)	108 (68.4%)	51 (52.0%)	
Shadowing	50 (19.5%)	18 (11.4%)	32 (32.7%)	
Enhancement	36 (14.1%)	26 (16.5%)	10 (10.2%)	
Combined	7 (2.7%)	2 (1.3%)	5 (5.1%)	
Not applicable	4 (1.6%)	4 (2.5%)	0 (0.0%)	
Calcifications				0.012*
No	225 (87.9%)	145 (91.8%)	80 (81.6%)	
In a mass	23 (9.0%)	7 (4.4%)	16 (16.3%)	
Not applicable	4 (1.6%)	4 (2.5%)	0 (0.0%)	
Intraductal	2 (0.8%)	1 (0.6%)	1 (1.0%)	
Indefinable	2 (0.8%)	1 (0.6%)	1 (1.0%)	

BMI, body mass index; OR, odds ratio. *P < 0.05.

### Model performance comparison and feature visualization

To ensure strict evaluation without data leakage, a rigorous comparison was conducted between the proposed method and baseline strategies using an OOF prediction strategy across the 5-fold cross-validation. As shown in Table 2, the Baseline (image-only) model achieved an AUC of 0.795 with a sensitivity of 0.408, highlighting the challenge of relying purely on visual features. To isolate the contribution of clinical semantics, we evaluated a Clinical baseline, which yielded a strong AUC of 0.890, confirming that objective morphological descriptors are powerful predictors. The Concat Fusion strategy improved the visual model’s AUC to 0.883, confirming the benefit of multimodal integration, though it still fell short of the strong Clinical prior. To investigate the impact of our cross-attention mechanism and transfer learning, we evaluated the CGA-Net. The pre-trained CGA-Net achieved the highest overall ranking capability, reaching a peak AUC of 0.915. Notably, the CGA-Net achieved the best-balanced clinical performance at the standard 0.5 decision threshold. This default probability threshold was deliberately maintained without post-hoc optimization (such as maximizing Youden’s Index) to objectively evaluate the model’s native calibration and strictly avoid threshold-selection bias. It yielded the highest overall accuracy of 0.857. While the image-only baseline exhibited a higher absolute specificity (0.916) solely due to heavily biased under-prediction of malignancy (sensitivity 0.408), the from-scratch CGA-Net achieved an optimally balanced specificity of 0.831 while maintaining an excellent sensitivity of 0.898. Compared to the pre-trained version, the from-scratch model significantly reduced false positives (improving specificity from 0.779 to 0.831). This indicates that direct guidance from objective clinical semantics allows the network to learn highly discriminative, task-specific representations without relying on external pre-training priors. This comparison between Concat Fusion and CGA-Net serves as a critical ablation study, demonstrating that active semantic querying is fundamentally superior to passive feature concatenation for this multimodal diagnostic task.

**TABLE 2:** Quantitative comparison of diagnostic performance among different deep learning models based on aggregated Out-Of-Fold (OOF) predictions.

Methods	Modality	Fusion strategy	Initialization	AUC (95% CI)	Accuracy	Sensitivity	Specificity
Baseline	Image	–	ImageNet	0.795(0.736–0.846)	0.718(0.663–0.770)	0.408(0.309–0.509)	0.916(0.871–0.959)
Clinical	Clinical	–	Random	0.890(0.844–0.928)	0.817(0.766–0.861)	0.847(0.770–0.917)	0.799(0.736–0.857)
Concat Fusion	Img + Clin	Concatenation	ImageNet	0.883(0.840–0.921)	0.798(0.750–0.845)	0.622(0.526–0.716)	0.909(0.863–0.953)
CGA–Net(Pre-trained)	Img + Clin	Cross-Attention	Pre-trained	0.915(0.876–0.944)	0.825(0.778–0.869)	0.898(0.837–0.950)	0.779(0.712–0.842)
CGA-Net	Img + Clin	Cross-Attention	From Scratch	0.905(0.863–0.941)	0.857(0.810–0.901)	0.898(0.837–0.954)	0.831(0.768–0.890)

AUC, Area Under the Receiver Operating Characteristic Curve; CI, Confidence Interval; CGA-Net, Cross-attention Guided Network.

**FIGURE 2:**
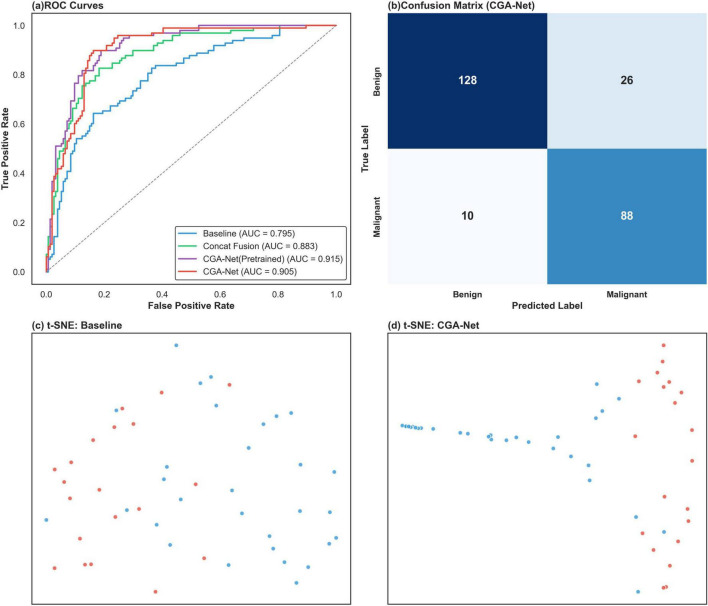
**(a)** ROC curves comparing CGA-Net against Baseline and Pre-trained models based on the aggregated Out-Of-Fold (OOF) predictions across the entire dataset. **(b)** Confusion matrix of CGA-Net showing high sensitivity. **(c)** t-SNE visualization of the Baseline model features showing class overlap. **(d)** t-SNE visualization of CGA-Net features showing clear separation between benign and malignant clusters. t-SNE, t-Distributed Stochastic Neighbor Embedding.

The receiver operating characteristic (ROC) curves (Figure 2a) illustrate the comparative performance of the four evaluated models across the entire dataset of 252 patients. The Baseline (image-only) model struggled with an AUC of 0.795, while the Concat Fusion strategy improved the AUC to 0.883. The cross-attention mechanism proved highly effective, with the CGA-Net (Pre-trained) achieving the highest overall ranking capability (AUC 0.915, *P* < 0.0001 vs. Baseline), closely followed by the CGA-Net (AUC 0.905, *P* < 0.001 vs. Baseline, and *P* = 0.417 vs. Clinical baseline). Although the AUC improvement over the strong Clinical baseline was not statistically significant, CGA-Net demonstrated superior overall accuracy and provided essential visual explainability—a critical advantage over pure clinical models in real-world diagnostic workflows. The global confusion matrix (Figure 2b) further demonstrates the clinical viability of the from-scratch CGA-Net at the standard 0.5 threshold. Out of the 252 cases, the model correctly identified 88 out of 98 malignant lesions and 128 out of 154 benign lesions, resulting in only 10 missed malignancies (false negatives) and 26 false alarms (false positives). This achieves an excellent balance between sensitivity (89.8%) and specificity (83.1%). t-Distributed Stochastic Neighbor Embedding (t-SNE) visualizations (Figures 2c,d) on a representative fold illustrate the underlying feature distributions: while Baseline features exhibit significant “Class Overlap,” CGA-Net generates “Clear Separation” between benign (blue) and malignant (red) clusters, proving the efficacy of semantic guidance. To verify statistical significance and ensure no data leakage occurred during evaluation, a bootstrap analysis with 1,000 iterations was performed on the aggregated predictions. The resulting 95% confidence interval for CGA-Net was [0.863, 0.941], confirming the robust internal validity of the results.

### Clinical utility and confidence calibration

**FIGURE 3:**
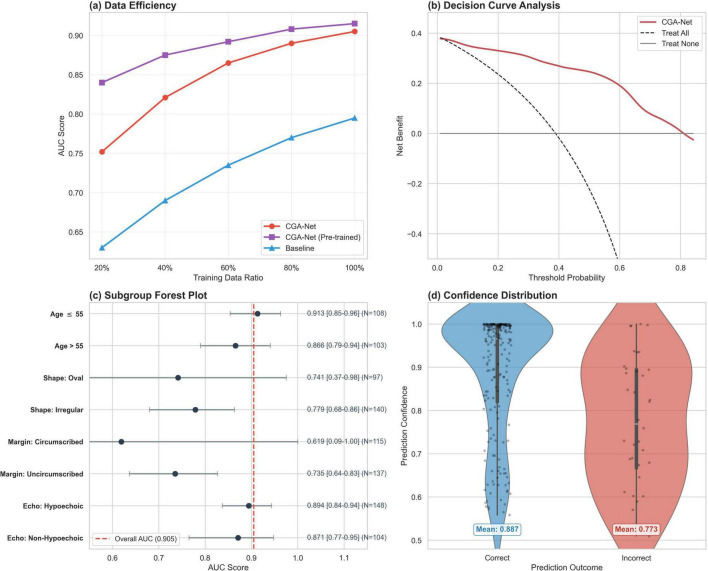
**(a)** Comparison of AUC scores across varying training data ratios for CGA-Net, pre-trained CGA-Net, and baseline models. **(b)** Decision curve analysis illustrating the net clinical benefit of the model compared with “Treat All” and “Treat None” strategies. **(c)** AUC performance across subgroups stratified by tumor margin, shape, and patient ages. **(d)** Violin plots showing the distribution of prediction confidence for correct versus incorrect results.

The clinical translational potential of CGA-Net was evaluated through data efficiency, decision utility, subgroup robustness, and confidence calibration (Figure 3). As shown in Figure 3a, while the pre-trained model exhibited a higher starting baseline in low-data regimes, the CGA-Net trained from scratch demonstrated rapid learning capability. Directed strictly by clinical semantics, its performance scaled steeply and converged to a highly competitive OOF AUC of 0.905 using the full dataset. Decision Curve Analysis (DCA) in Figure 3b indicates that the CGA-Net-guided strategy provides a substantial net clinical benefit over standard “treat-all” or “treat-none” schemes across a wide range of clinically relevant threshold probabilities, with the curve distinctly elevated above baselines. To rigorously assess generalizability, a subgroup forest plot analysis (Figure 3c) was conducted. The model exhibited exceptional stability across diverse patient demographics and morphological profiles, maintaining consistently high AUCs with robust 95% confidence intervals even in challenging subgroups, such as non-hypoechoic lesions or uncircumscribed margins. Finally, Figure 3d presents the confidence calibration results. Quantitative calibration analysis of the OOF predictions yielded an Expected Calibration Error (ECE) of 0.0993 and a Brier score of 0.1175, indicating highly reliable probability estimates. For correctly classified cases, the model yielded a high average confidence (Mean = 0.887), whereas for misclassified cases, the confidence dropped significantly (Mean = 0.773) with higher variance. This clear separation is highly valuable for real-world clinical workflows, allowing the system to automatically flag uncertain predictions for secondary expert review.

### Interpretability and system prototyping

**FIGURE 4:**
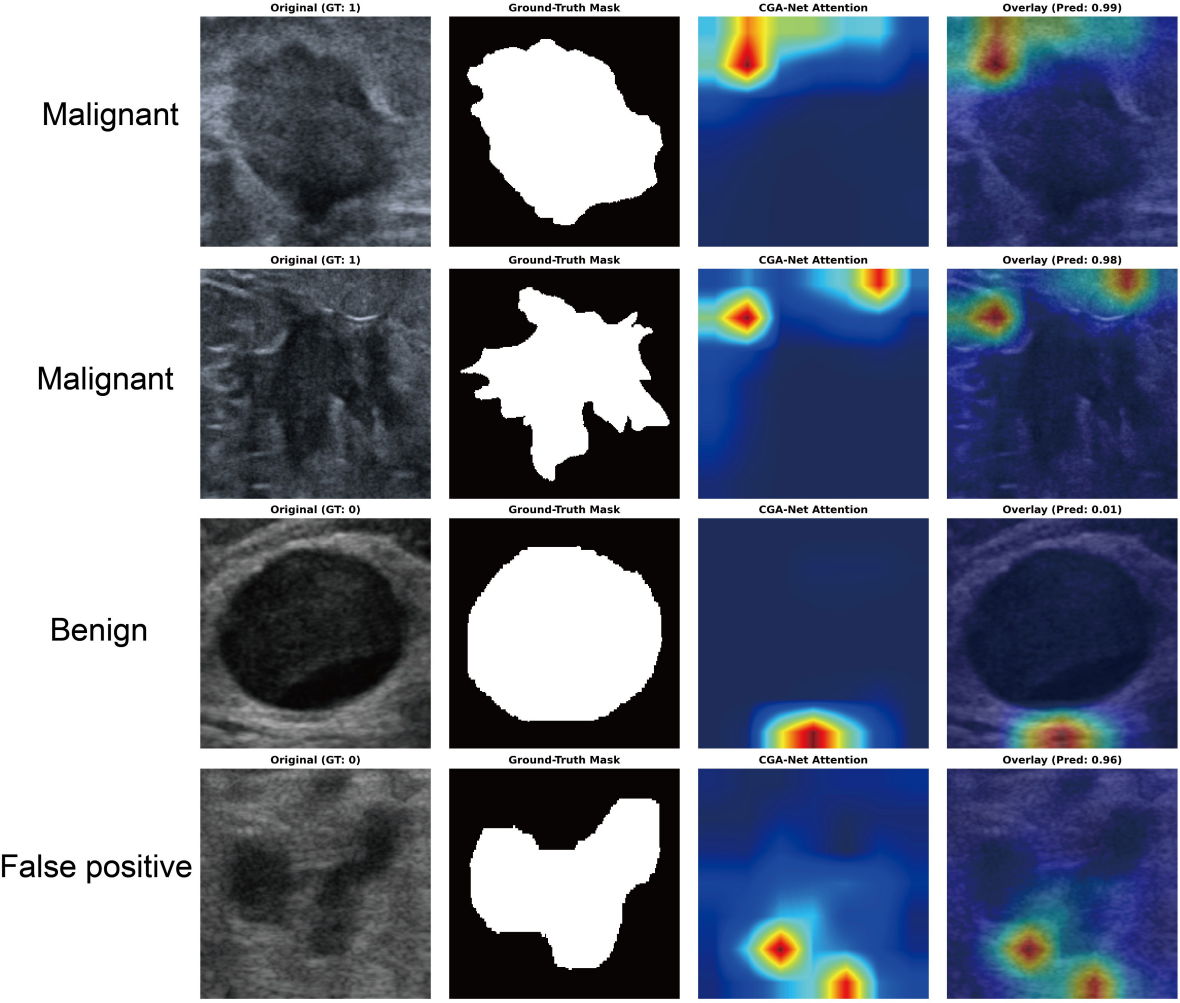
Model interpretability using attention maps. Two representative malignant cases (top two rows, demonstrating direct boundary overlap and peripheral tissue focus, respectively), a benign case (third row), and a false positive case (bottom row) are shown. Each example includes the original ultrasound image, the corresponding ground-truth mask, the generated attention heatmap, and the overlay with predicted probability (Pred). Warmer colors indicate regions of higher diagnostic importance. GT, ground truth.

**FIGURE 5:**
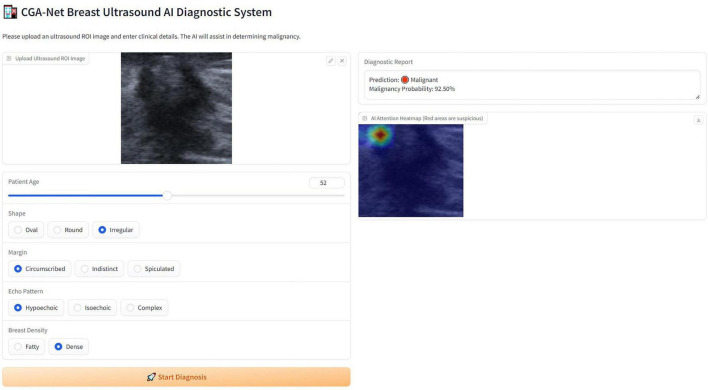
Interface of the clinical decision support system. The prototype integrates visual and semantic inputs to provide explainable diagnosis. By uploading an ultrasound ROI image and selecting corresponding clinical descriptors, the user receives a probability score and an attention map overlay generated strictly within the ROI boundaries. This transparent output assists clinicians in validating whether the AI’s prediction aligns with pathological tumor features.

Ideally, a clinical AI system should provide not only accurate predictions but also transparent visual evidence to facilitate clinician trust. Inspired by recent advancements in interpretable CAD systems, to verify the pathological consistency of CGA-Net, we visualized the attention maps directly generated by the cross-attention module, presenting the original ultrasound images alongside their corresponding ground-truth (GT) masks for direct visual comparison (23). As illustrated in Figure 4, the heatmap distribution dynamically adapts to the most discriminative features of each specific case. Because the input images are pre-cropped ROIs where the lesion is already centrally located, the model intelligently shifts its semantic focus rather than merely locating the tumor center. For instance, in the first malignant case (Figure 4, top row), the high-attention region successfully overlaps with the ground-truth mask, directly pinpointing the highly irregular and spiculated boundaries of the tumor. Conversely, in the second malignant case (Figure 4, second row), the attention deliberately extends further into the tumor periphery and surrounding tissue—appearing partially outside the core mask. This behavior is highly consistent with clinical reasoning, as critical malignant features like posterior acoustic shadowing and angular margins manifest exactly at the intersection of the mass and healthy tissue. Because the cross-attention inherently operates on the deep, low-resolution (7×7) semantic feature maps from the ResNet backbone, these visualizations represent high-level regional focus rather than fine-grained pixel segmentation. For true-negative cases, the heatmaps show minimal activation, correctly indicating the absence of suspicious features. Nevertheless, qualitative error analysis exposes distinct failure modes. In false-positive instances (Figure 4, bottom), complex internal textures or pronounced shadowing in benign lesions, such as degenerating fibroadenomas, provoke spurious attention activations. Conversely, false-negative errors primarily involve atypical malignancies like mucinous carcinomas, which frequently display deceptively well-circumscribed margins and posterior enhancement. Lacking classical malignant semantic features, the cross-attention module is misdirected and subsequently underestimates the risk of malignancy. Explicitly defining these limitations ensures clinicians understand the boundary conditions for integrating this tool with their independent judgment. Building upon these algorithmic validations, we developed a web-based prototype of the CAD system to demonstrate clinical feasibility (Figure 5). To ensure strict alignment with our training pipeline, the interface requires clinicians to upload pre-cropped ROI images—or manually crop the lesion from the full scan within the tool—alongside the structured clinical descriptors. Upon analysis, the system outputs a real-time malignancy probability along with an overlay of the attention heatmap, which is generated exclusively within the defined ROI boundaries. This transparent visual feedback serves as a “second opinion,” allowing radiologists to rapidly verify whether the AI’s high-confidence prediction is substantiated by suspicious morphological features, thereby enhancing diagnostic confidence in clinical workflows.

## Discussion

In this study, we developed CGA-Net, a cross-attention-guided framework that synergizes clinical morphological semantics with ultrasound visual features. The principal finding of this work is that explicitly embedding structured clinical priors enables the model to surpass the performance ceiling of both pure computer vision and isolated clinical semantic approaches. Evaluated under a rigorous OOF protocol, our pre-trained CGA-Net achieved a highly competitive overall AUC of 0.915, substantially outperforming the image-only baseline (AUC 0.795) as well as the robust clinical baseline (AUC 0.890). This comparison reveals a critical insight: while morphological descriptors are dominant predictors of malignancy, relying solely on them creates a performance bottleneck due to the discrete nature of categorical variables and potential operator variability. CGA-Net overcomes this limitation by leveraging visual features as a continuous “safety net,” compensating for textual ambiguity to achieve superior stability. Interestingly, our results reveal a nuanced trade-off regarding transfer learning. While the pre-trained CGA-Net achieved the highest overall ranking capability (AUC 0.915), the model trained from scratch achieved the best-balanced clinical performance at the standard diagnostic threshold, yielding the highest overall accuracy (0.857) and specificity (0.831). This suggests that the strong semantic guidance provided by clinical descriptors effectively acts as a form of “conceptual pre-training.” By training from scratch, the cross-attention mechanism forces the visual encoder to exclusively learn features that are tightly correlated with objective clinical descriptors, minimizing domain-specific noise from external visual weights and effectively reducing false positive diagnoses.

By employing an OOF evaluation and explicitly excluding subjective BI-RADS scores, we ensured the model’s robust performance is driven strictly by objective morphological patterns. Our statistical analysis (Table 1) revealed that 96.9% of malignant cases exhibited non-circumscribed margins, a feature strongly correlated with pathological infiltration (24). CGA-Net effectively learned this “pathological truth” via the cross-attention mechanism, mapping semantic queries, such as “irregular margin,” to corresponding visual regions. This implies that the model is not “memorizing” cases, but rather mastering the same diagnostic criteria used by senior radiologists, where specific morphological combinations yield positive predictive values exceeding 90% (25).

A critical consideration in this framework is the reliance on manually extracted clinical descriptors. One might argue that relying on human-defined inputs reintroduces the very subjectivity that AI aims to eliminate. However, we argue that CGA-Net functions not as a replacement for radiologist perception, but as an objective “reasoning engine.” By treating clinical descriptors as semantic queries, the model mathematically aligns these standardized priors with objective pixel-level evidence (raw ultrasound images), thereby standardizing the decision-making boundary and reducing the variability inherent in purely subjective diagnosis (6, 26).

Recent literature has seen remarkable progress in transformer-based and multimodal breast CAD systems. For instance, advanced frameworks like ETECADx and hybrid residual transformers utilize self-attention mechanisms (Vision Transformers) on convolutional backbones to achieve exceptional accuracy on digital mammograms (27, 28). Furthermore, sophisticated multimodal systems have successfully fused mammograms with ultrasound images using ensemble CNNs and ViT attention, incorporating post-hoc tools like Grad-CAM for visual explainability (29). While these state-of-the-art models excel in extracting global visual dependencies and cross-imaging features, they predominantly operate within the purely visual domain. CGA-Net addresses a distinct methodological gap by performing cross-domain fusion—integrating raw visual data directly with objective clinical semantics. From an architectural perspective, our framework utilizes standard components (ResNet, MLP, Cross-Attention). However, its conceptual novelty addresses a critical bottleneck in multimodal CAD. As demonstrated by our ablation results (Concat Fusion AUC 0.883 vs. CGA-Net AUC 0.905), simple feature concatenation passively merges domains, making it susceptible to visual background noise. In contrast, our semantic-guided cross-attention actively treats clinical descriptors as high-level queries, dynamically filtering the visual feature space to retain only the pixel evidence that correlates with the objective morphological priors. Moreover, unlike *post-hoc* explainability methods, CGA-Net’s attention maps are intrinsically generated by this clinical-visual cross-attention, ensuring the model’s visual focus is inherently tied to established pathological criteria. This design offers a meaningful advancement in building trustworthy and transparent multimodal frameworks.

Beyond classification metrics, the “black-box” nature of deep learning remains a barrier to clinical trust (6). Unlike conventional CNNs that may erroneously focus on background artifacts (30), CGA-Net demonstrates transparent and logically consistent visual attention. As evidenced in our visualization results, the model exhibits a “positive-attention” mechanism for malignant cases, regionally focusing on areas containing spiculations and micro-lobulations rather than delineating exact pixel boundaries. More importantly, it demonstrates a “negative-reasoning” capability for benign cases, characterized by a lack of strong attention activation. This mirrors the cognitive process of a radiologist: a benign diagnosis is often established by the absence of suspicious features rather than the presence of specific benign markers (31). This alignment between algorithmic attention and human cognitive focus significantly enhances the explainability and trustworthiness of the system. Furthermore, because the attention maps are mathematically bound to deterministic clinical queries rather than unconstrained visual features, this semantic-guided mechanism inherently ensures high cross-fold interpretability consistency, preventing random feature drift across different data splits.

The clinical translational value of CGA-Net is further supported by its robustness and confidence awareness. Deep learning models often suffer from performance degradation when applied to subpopulations or limited data (32). However, our model demonstrated strong data efficiency, with performance scaling rapidly and stabilizing as training data increased (Figure 3a). To rigorously assess generalizability, a subgroup forest plot analysis was conducted (Figure 3c). The model exhibited exceptional stability across diverse patient demographics and morphological profiles, maintaining robust AUCs and reliable 95% confidence intervals even in challenging subgroups such as uncircumscribed margins or non-hypoechoic lesions. This suggests that the multi-modal fusion strategy effectively mitigates the bias often seen in image-only models. Crucially, the clear separation of confidence scores between correct and incorrect predictions enables the implementation of “human-in-the-loop” workflows (26). For correctly classified cases, the model showed high confidence (Mean = 0.887), whereas for misclassified cases, the confidence dropped significantly (Mean = 0.773) with higher variance. High-confidence predictions can facilitate rapid triage, while low-confidence outputs can automatically trigger expert review, thereby balancing efficiency with patient safety.

Despite these promising findings, our study has limitations. First, this is a retrospective study limited to internal validation. Because the external public datasets utilized in this study lacked paired clinical reports and were exclusively used for visual pre-training, our framework has not yet undergone true external multimodal validation. Consequently, claims regarding its broad generalizability must be interpreted with caution. Multi-center external validation on datasets containing both ultrasound images and comprehensive clinical reports is essential to definitively test its true clinical robustness across different hospital systems and varied operator experiences (33). Second, the current framework relies on structured text inputs extracted from reports. This requires a structured reporting environment, which may not be available in all clinical settings. Future work will focus on integrating Natural Language Processing (NLP) modules, such as Large Language Models (LLMs), to automatically extract semantic features from unstructured clinical notes, creating a fully end-to-end automated pipeline (34). Third, the proposed pipeline currently relies on binary masks to extract the ROI prior to classification. As pointed out, this strategy provides the model with privileged lesion localization information. While this approach was deliberately chosen to isolate and validate the pure classification power of our cross-attention fusion mechanism without the confounding variable of background localization errors, it may artificially simplify the task. Consequently, the performance metrics reported herein reflect the model’s capability given perfect localization, which is likely inflated compared to a true real-world setting using full, uncropped ultrasound images. In real-world clinical environments, manual ROI segmentation is time-consuming and limits high-throughput applicability. To operate effectively on full ultrasound images and mitigate this localization bias, this manual step must be replaced by an automated lesion detection module, such as You Only Look Once (YOLO) variants, as successfully demonstrated in recent end-to-end computer-aided diagnosis frameworks (29). While our current prototype demonstrates a semi-automated workflow where the lesion is manually localized, integrating a robust automated detection model to create a fully end-to-end diagnostic pipeline is a critical focus for our future translational efforts.

## Conclusion

In conclusion, we proposed CGA-Net, a cross-attention-guided multi-modal framework for breast ultrasound diagnosis. By dynamically fusing objective morphological semantics with visual features, our framework achieves robust diagnostic performance and superior data efficiency. Evaluated under a rigorous OOF protocol, the CGA-Net trained from scratch demonstrated the most balanced clinical utility, yielding a robust OOF AUC of 0.905 alongside the highest overall accuracy (0.857) and an optimal balance of sensitivity and specificity (0.831). Additionally, the pre-trained CGA-Net reached a peak overall ranking AUC of 0.915. Coupled with transparent visual explainability and reliable confidence calibration, CGA-Net serves as a trustworthy “second opinion” tool to standardize decision-making, reduce diagnostic variability, and ultimately minimize unnecessary biopsies.

## Data Availability

The original contributions presented in this study are included in this article/supplementary material, further inquiries can be directed to the corresponding authors.
